# Supplementary figures and images for: Ribosome profiling reveals downregulation of UMP biosynthesis as the major early response to phage infection
Source: Microbiol Spectr. 2024 Mar 7;12(4):e03989-23. doi: 10.1128/spectrum.03989-23 (PMC10986495; doi:10.1128/spectrum.03989-23)

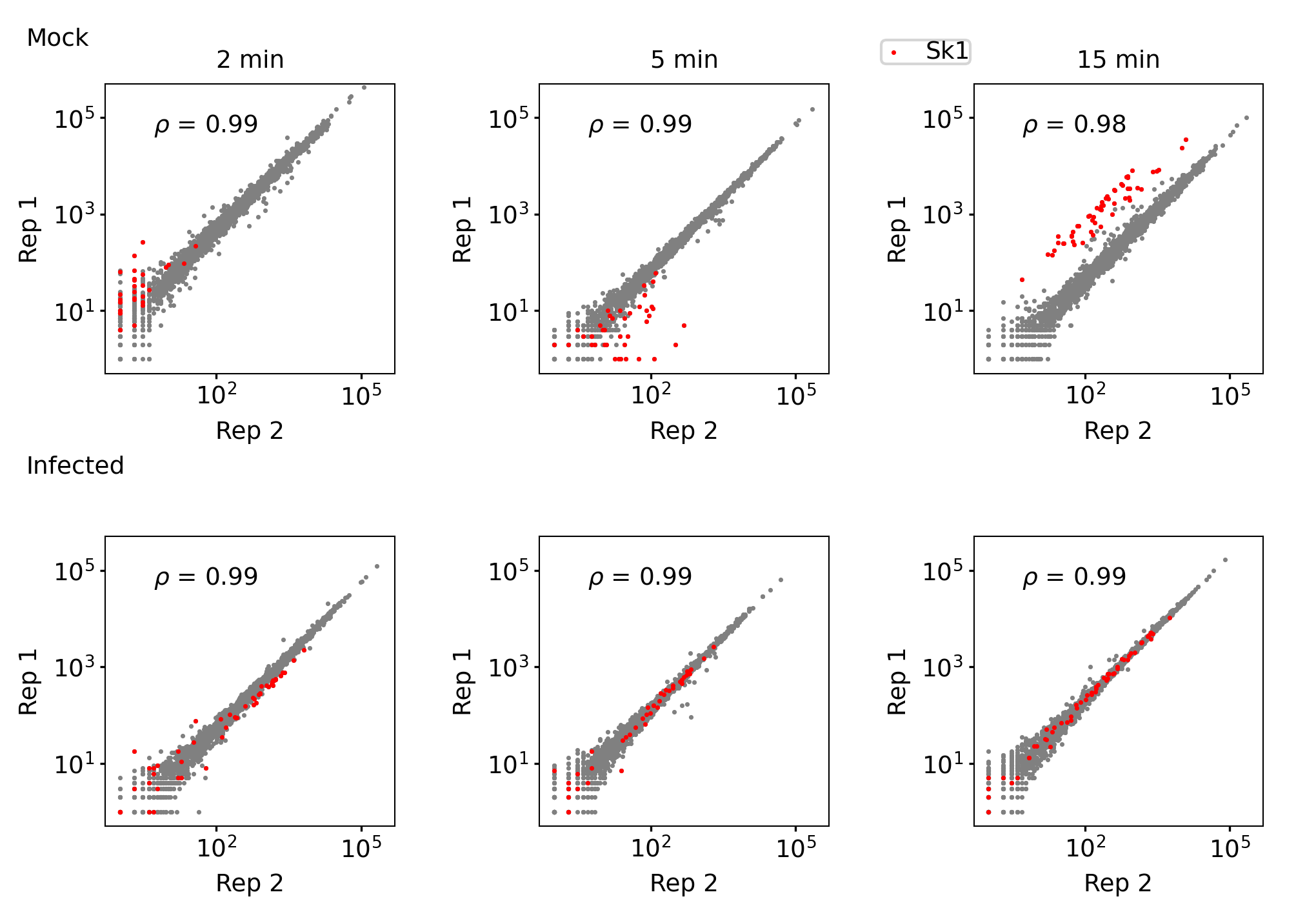

Supplement: Fig. S1 — Comparison of the number of mapped reads per gene across replicates. [file spectrum.03989-23-s0001.tiff]

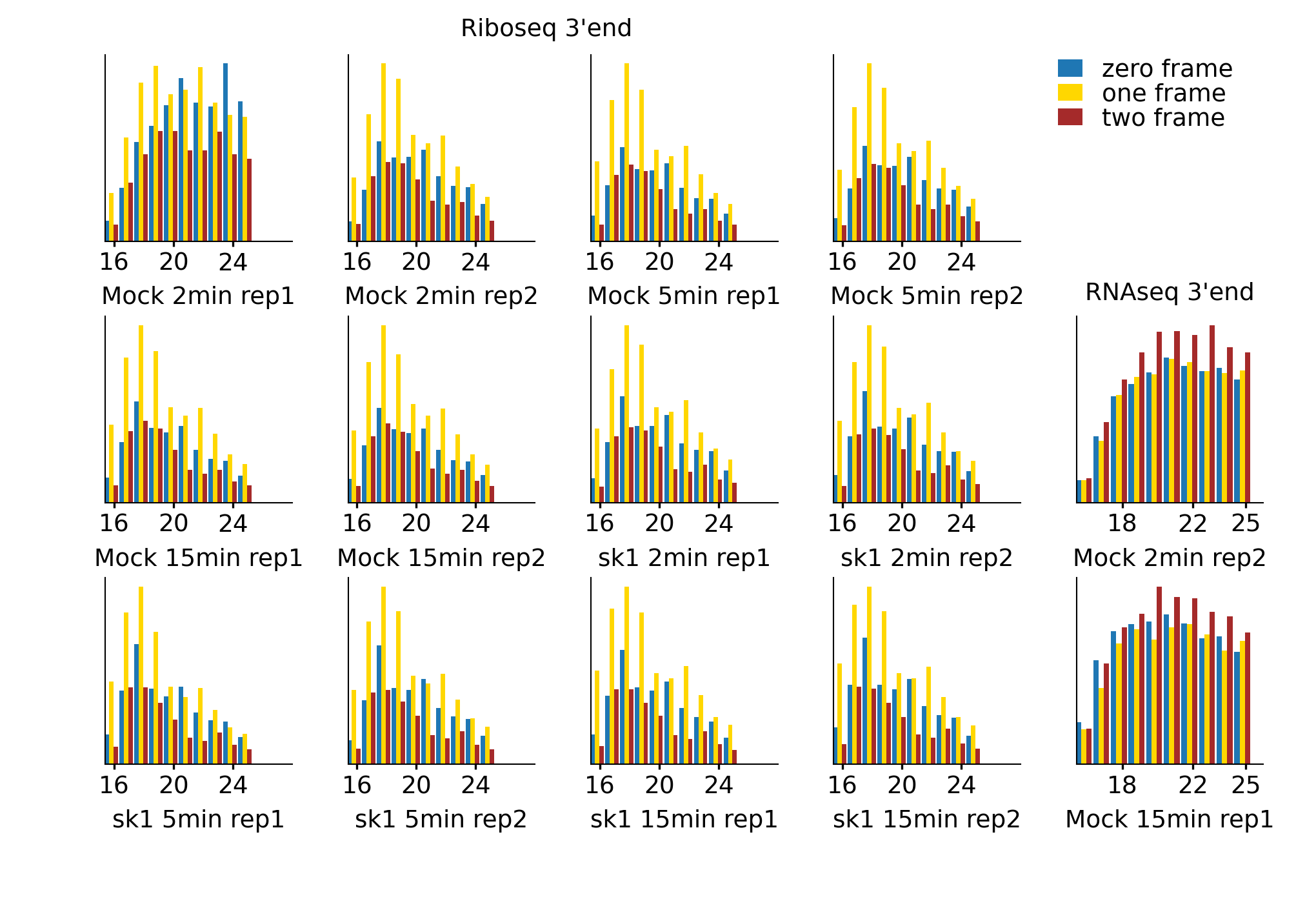

Supplement: Fig. S2 — Subcodon periodicity signal distribution obtained across all Ribo-seq and RNA-seq samples. [file spectrum.03989-23-s0002.tiff]

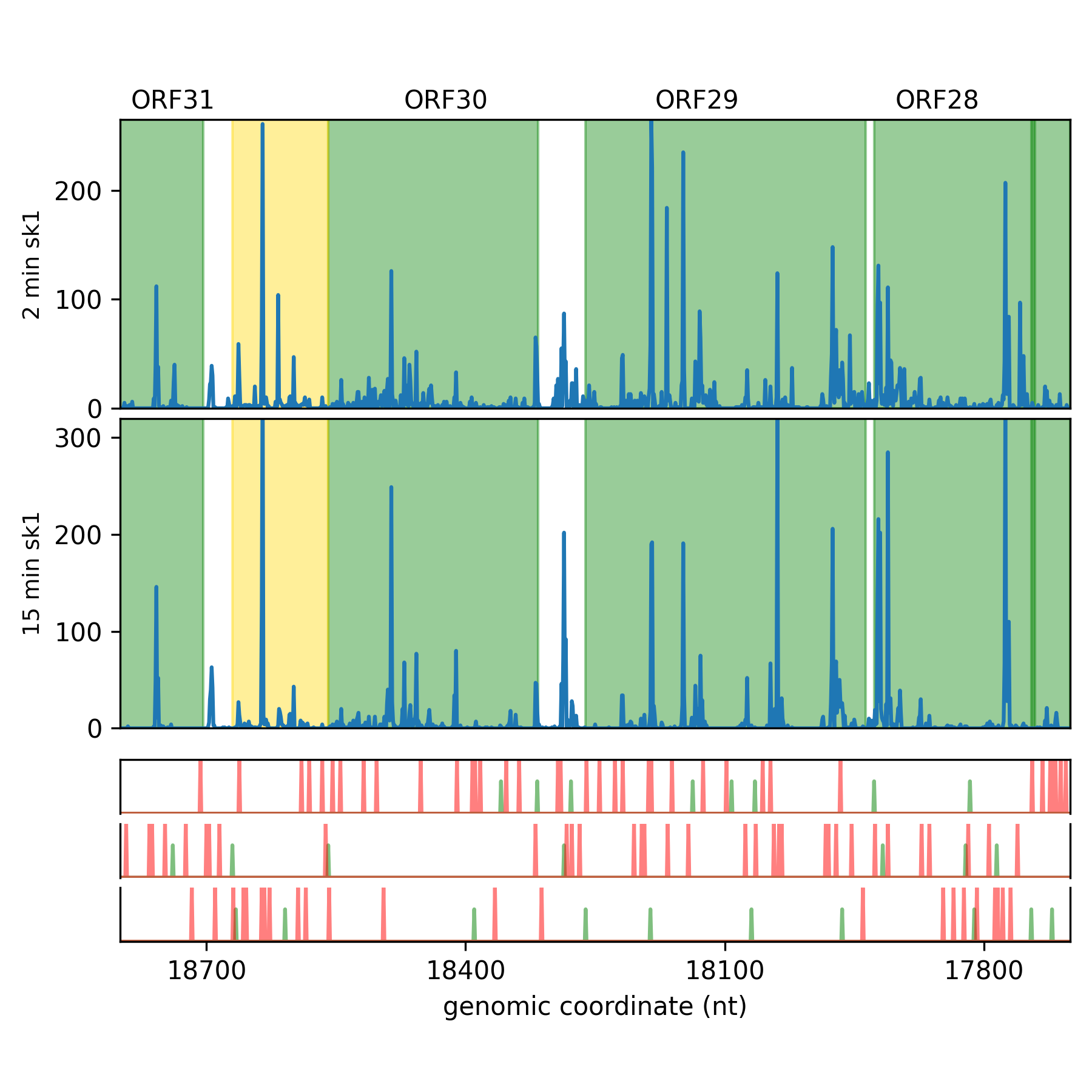

Supplement: Fig. S3 — New translated ORF found in sk1. [file spectrum.03989-23-s0003.tiff]

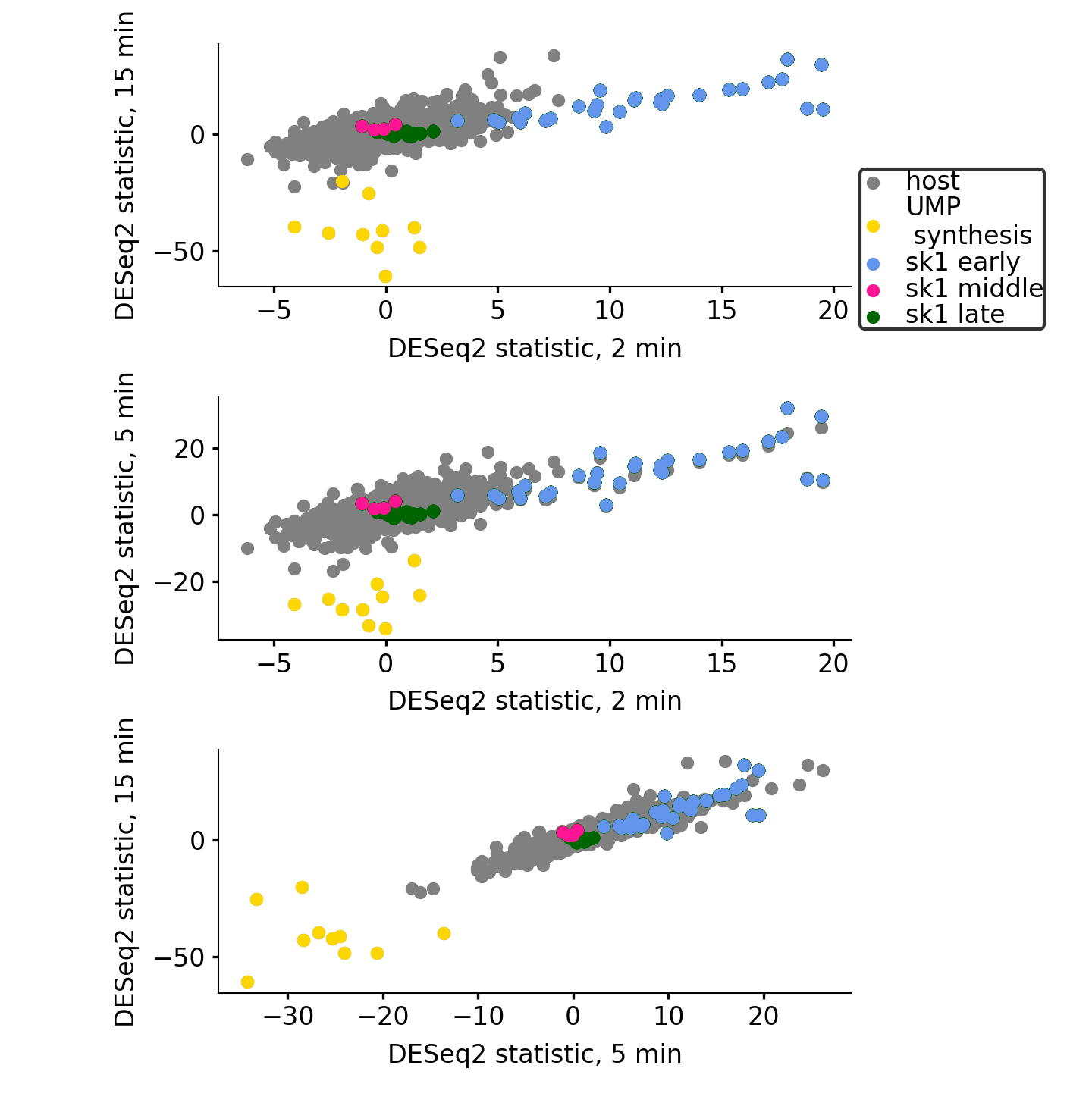

Supplement: Fig. S4 — Gene expression response consists of a major shift within 2 minutes p.i. [file spectrum.03989-23-s0004.tiff]

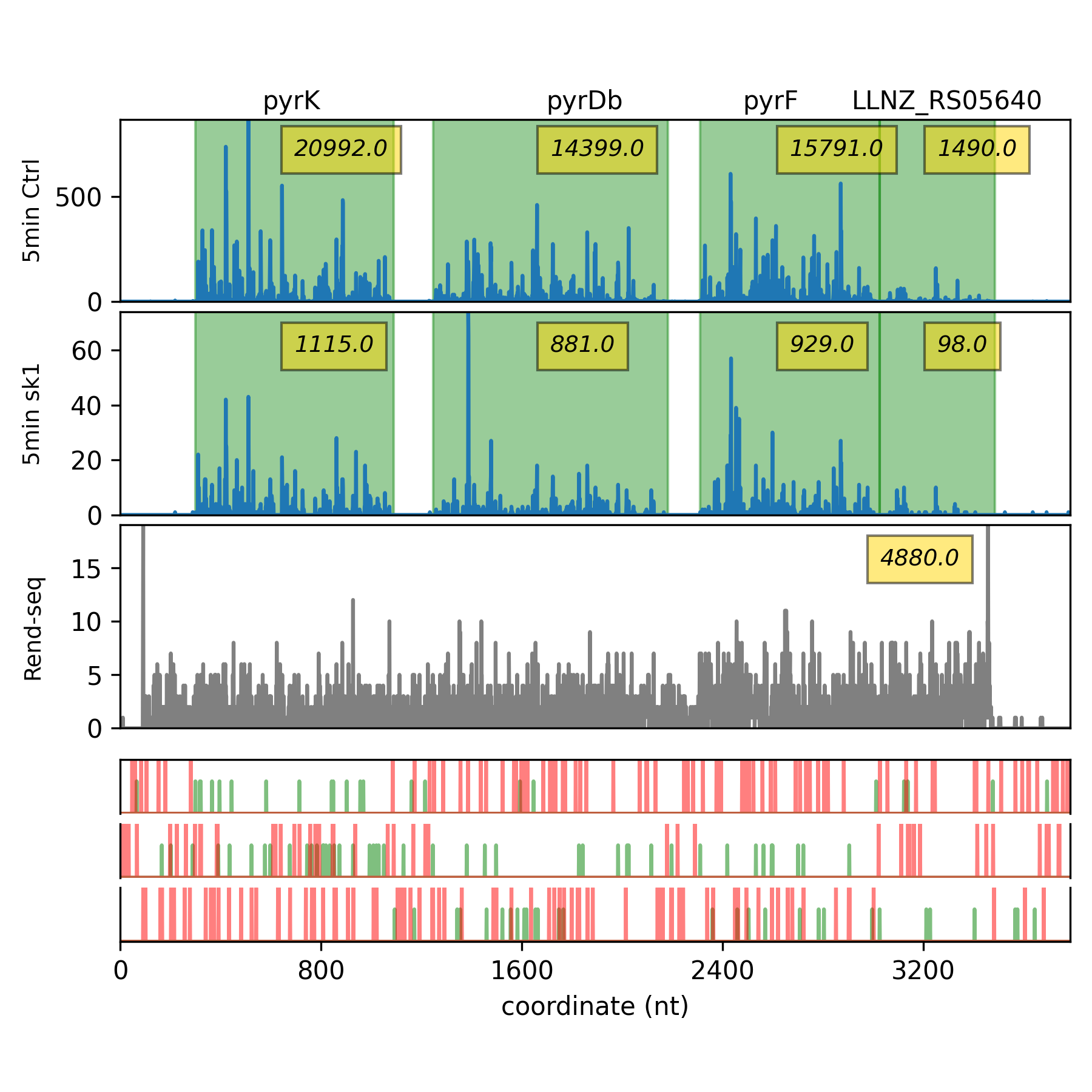

Supplement: Fig. S5 — LLNZ_RS05640/orfC confirmed to belong to the pyrKDbForfC operon. [file spectrum.03989-23-s0005.tiff]

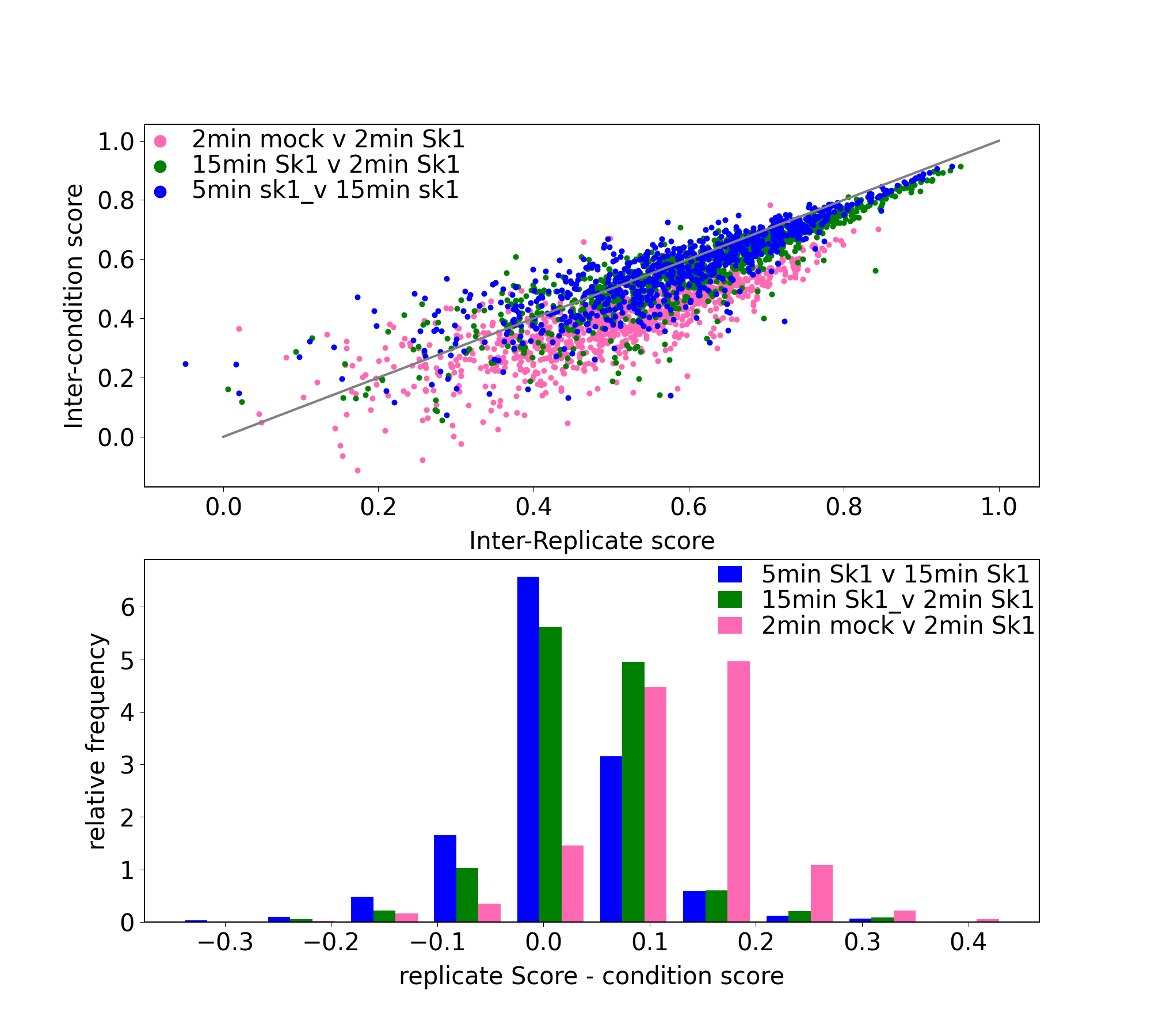

Supplement: Fig. S6 — Changes in ribosome profile silhouettes occur mostly within 2 minutes p.i. [file spectrum.03989-23-s0006.tiff]

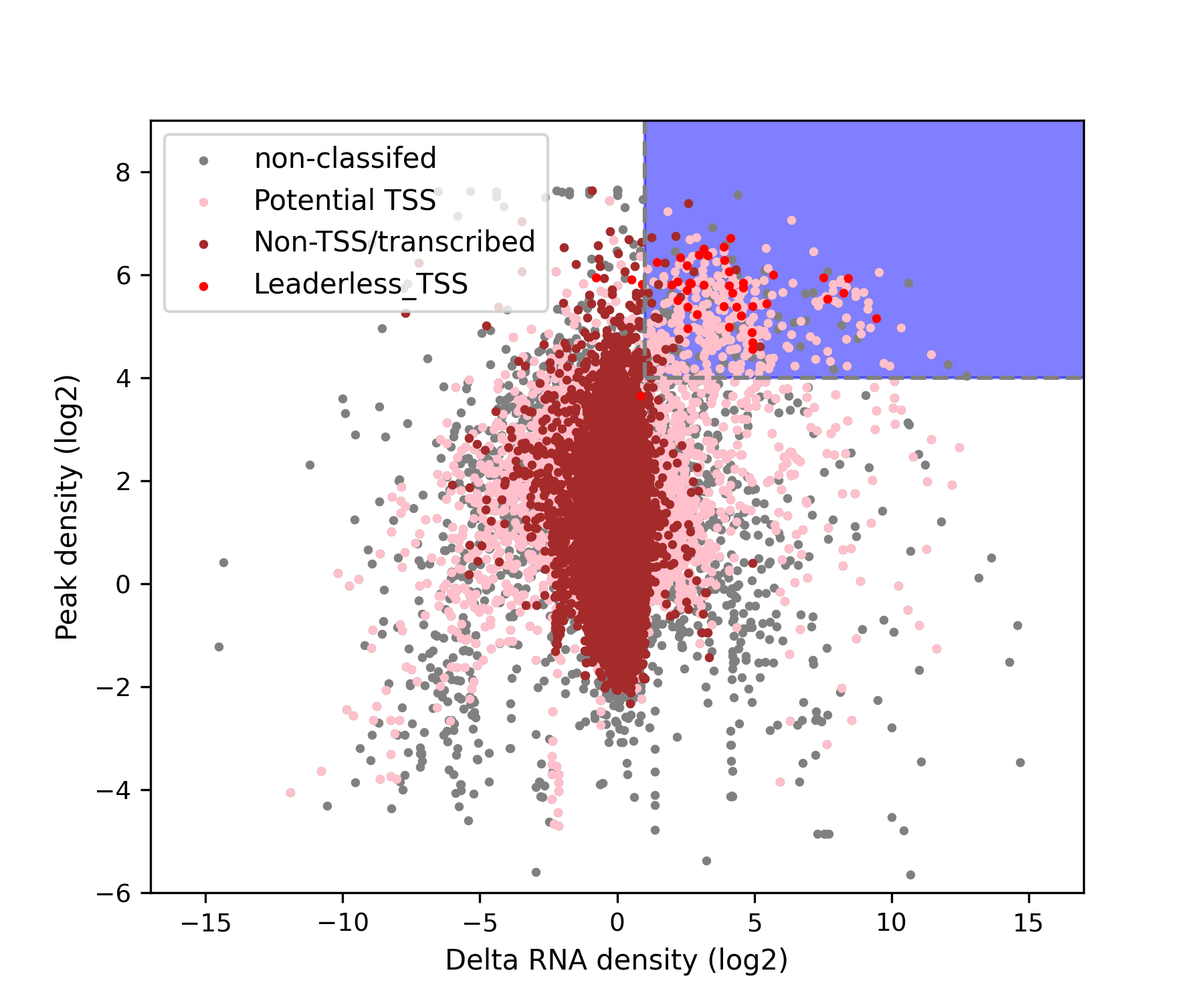

Supplement: Fig. S7 — Identification of transcriptional start sites. [file spectrum.03989-23-s0007.tiff]
