# Supplementary material for: Ribosome profiling reveals downregulation of UMP biosynthesis as the major early response to phage infection
Source: Microbiol Spectr. 2024 Mar 7;12(4):e03989-23. doi: 10.1128/spectrum.03989-23 (PMC10986495; doi:10.1128/spectrum.03989-23)
Supplement: Supplemental legends — Legends for Fig. S1 to S7. [file spectrum.03989-23-s0008.docx]

**Figure S1. Comparison of the number of mapped reads per gene across replicates.** Number of mapped reads per gene across replicates for Ribo-seq samples at 2-, 5-, and 15-minutes p.i. Reads aligning to the sk1 genes are indicated in red. The Spearman’s correlation coefficient is indicated.

**Figure S2**. **Subcodon periodicity signal distribution obtained across all Ribo-seq and RNA-seq samples.** The 3’end of the read was used to indicate the position of the read. This produces a consistent phasing across various read lengths in the Ribo-seq samples.

**Figure S3. New translated ORF found in sk1.** The top two panels show unambiguously mapped reads to part of sk1 genome encoding early genes. Previously annotated ORFs are in green while the novel ORF is indicated in gold. The bottom panel is an open reading frame organisation plot, AUG and stop codon (UGA, UAG, UAG) occurrences in all three potential reading frames are indicated with green and red bars, respectively.

**Figure S4. Gene expression response consists of a major shift within 2 minutes p.i.** Subpanels show the pairwise comparisons of DESeq2 test statistics obtained across different time-points. The high similarity of test statistics at 5- and 15 minutes timepoints reveals a modest after the 5 minute p.i. timepoint.

**Figure S5. LLNZ_RS05640/orfC confirmed to belong to the *pyrKDbForfC* operon.** LLNZ_RS05640 is the fourth (rightmost) gene and was classified as a hypothetical gene. The gene is found to be translated and is repressed upon infection like the other genes in the operon. Combined Rend-seq and RNA-seq data are provided in the third panel (from top). The number of mapped reads is indicated. The bottom panel is an open reading frame organisation plot, AUG and stop codon (UGA, UAG, UAG) occurrences in all three potential reading frames are indicated with green and red bars, respectively.

**Figure S6. Changes in ribosome profile silhouettes occur mostly within 2 minutes p.i.** The upper panel presents a comparison of individual gene silhouettes within replicates (replicate score) and within different states (condition score). The lower panel depicts the distribution of the difference between the replicate and condition scores. The ribosome silhouettes of sk1 infected samples at 2 minutes p.i. exhibit a closer similarity to the sk1 samples at 15 minutes p.i. (blue) than to the corresponding mock-infected sample at 2 minutes p.i. (pink).


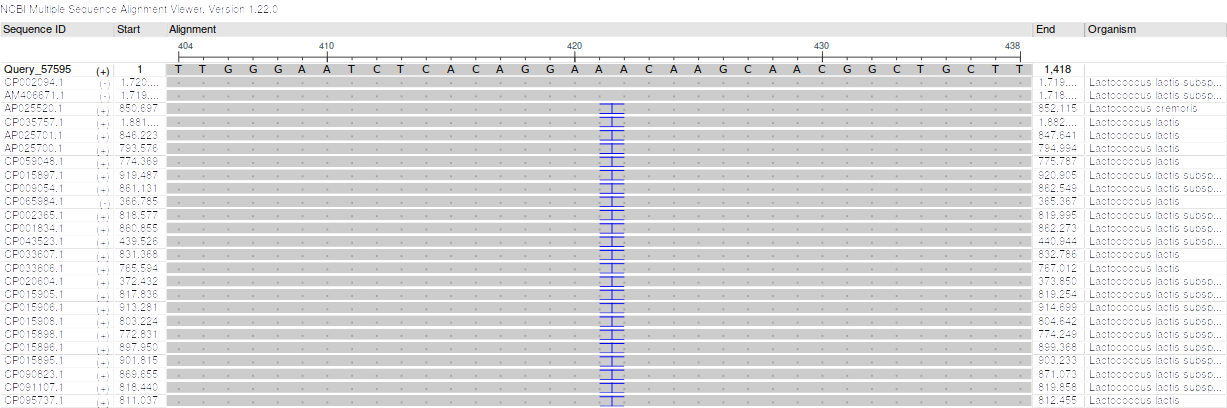
**Figure S7. Identification of Transcriptional Start Sites**. TSS were identified based on high RNA read density relative to background (x-axis) and increased ribosome footprint read density downstream i.e., Peak density (y-axis) (see Methods). Previously annotated leaderless TSS (red) were used as a “true positives set” while positions within annotated CDS (maroon) were a “true negative” in order to estimate the parameters of Peak density and RNA density which would be indicative of a TSS. The genomic positions that exceed both the thresholds are within the blue shading.
